# Maternal alcohol binge-drinking in the first trimester and the risk of orofacial clefts in offspring: a large population-based pooling study

**DOI:** 10.1007/s10654-016-0171-5

**Published:** 2016-06-27

**Authors:** Lisa A. DeRoo, Allen J. Wilcox, Rolv T. Lie, Paul A. Romitti, Dorthe Almind Pedersen, Ronald G. Munger, Lina M. Moreno Uribe, George L. Wehby

**Affiliations:** 1 Department of Global Public Health and Primary Care, University of Bergen, Postboks 7804, 5020 Bergen, Norway; 2 Epidemiology Branch, National Institute of Environmental Health Sciences/NIH, Durham, NC USA; 3 Norwegian Institute of Public Health, Medical Birth Registry of Norway, Bergen, Norway; 4 Department of Epidemiology, College of Public Health, The University of Iowa, Iowa City, IA USA; 5 Epidemiology, Biostatistics and Biodemography, Institute of Public Health, University of Southern Denmark, Odense, Denmark; 6 Department of Nutrition, Dietetics, and Food Sciences, Utah State University, Logan, UT USA; 7 Department of Orthodontics, College of Dentistry and Dental Clinics, The University of Iowa, Iowa City, IA USA; 8 Department of Health Management and Policy, The University of Iowa, 105 River Street, Iowa City, IA 52242 USA

**Keywords:** Cleft lip, Cleft palate, Alcohol

## Abstract

Using individual participant data from six population-based case–control studies, we conducted pooled analyses to examine maternal alcohol consumption and the risk of clefts among >4600 infants with cleft lip only, cleft lip with cleft palate, or cleft palate only and >10,000 unaffected controls. We examined two first-trimester alcohol measures: average number of drinks/sitting and maximum number of drinks/sitting, with five studies contributing to each analysis. Study-specific odds ratios (ORs) were estimated using logistic regression and pooled to generate adjusted summary ORs. Across studies, 0.9–3.2 % of control mothers reported drinking an average of 5+ drinks/sitting, while 1.4–23.5 % reported drinking a maximum of 5+ drinks/sitting. Compared with non-drinkers, mothers who drank an average of 5+ drinks/sitting were more likely to deliver an infant with cleft lip only (pooled OR 1.48; 95 % confidence intervals 1.01, 2.18). The estimate was higher among women who drank at this level 3+ times (pooled OR 1.95; 1.23, 3.11). Ever drinking a maximum of 5+ drinks/sitting and non-binge drinking were not associated with cleft risk. Repeated heavy maternal alcohol consumption was associated with an increased risk of cleft lip only in offspring. There was little evidence of increased risk for other cleft types or alcohol measures.

## Introduction

Heavy maternal alcohol consumption is associated with fetal alcohol syndrome, characterized by distinctive facial dysmorphology, prenatal and postnatal growth restriction, and central nervous system and neurodevelopmental abnormalities [1]. The association between maternal alcohol consumption and individual congenital malformations, such as orofacial clefts, is less clear [2]. Binge-level drinking, usually defined as 5 or more drinks per sitting [3], may be particularly harmful to fetal development because it exposes the fetus to higher blood alcohol concentrations than does drinking the same amount of alcohol over a longer period of time [4].

The results of epidemiologic studies on alcohol consumption and clefts are difficult to summarize, in part due to differences in alcohol measures and different time points of reference across studies. Some studies examined the frequency of maternal alcohol drinking or drink totals (weekly or monthly) during pregnancy, but not drinking pattern. Among the three studies examining binge-level drinking of an average of 5 or more drinks per sitting [5–7], all found an increased risk of infant cleft lip with or without cleft palate compared with non-drinkers, and one also found an increased risk of cleft palate only [7]. Small numbers of women who drank at binge levels, however, have made it difficult to assess this association in many studies. We conducted a pooled analysis of individual-level data from 6 population-based studies (3 in the United States and 3 in Europe) to examine first-trimester maternal binge-level drinking and the risk of orofacial clefts in offspring.

## Materials and methods

### Studies

Data for these analyses came from an international consortium of studies examining risk factors for orofacial cleft malformations [8]. Studies were chosen using the following criteria: (1) population-based, (2) available environmental and lifestyle data, and (3) agreement (with ethical approval) to share individual-level data for pooled analysis. Six studies were identified, together providing a pooled sample of 5272 cases and 11,461 controls: the Danish National Birth Cohort (DNBC) [9], the Iowa Case–Control Study (Iowa) [10], the National Birth Defects and Prevention Study (NBDPS) [11], the Norway Facial Clefts Study (NCL) [12], the Norwegian Mother and Child Cohort (MoBa) [13], and the Utah Child and Family Health Study (Utah) [14] (Table 1). All were case–control studies (Iowa, NBDPS, NCL, Utah) or case–control studies nested within prospective cohort studies (DNBC, MoBa) with enrollment periods ranging from the late 1980s to the 2000s. Infants with clefts were identified in national medical birth registries (DNBC, MoBa), state birth defects surveillance systems (Iowa, NBDPS, Utah), or referrals from hospitals handling cleft repair surgeries (NCL). Control subjects without cleft malformations were randomly sampled from state birth certificates (Iowa, Utah), birth certificates or hospital logs (NBDPS), participants in the corresponding underlying cohort studies (DNBC, MoBa), or a medical birth registry (NCL). In each study, self-administered questionnaires, in-person interviews, or telephone interviews were used to collect information from mothers on demographic characteristics, medical history, cigarette smoking, alcohol consumption and other exposures during pregnancy. Each study received approval from a local institutional review board and all mothers provided informed consent.

**Table 1:** Characteristics of studies included in the pooled analysis of maternal first trimester alcohol consumption and infant cleft risk

Study; no. cases/no. controls; design	Period of Enrollment (Birth years)	Location	Case Ascertainment	Control Ascertainment	Mode of exposure ascertainment; timing of completion	Participation Rates	Selected characteristics of control mothers		
							Age range (mean)	Smoking first trimester %	Education <high school %
DNBC; 179/828; Case–control analysis nested within cohort	1998–2002	Denmark	Live births Danish Facial Cleft Registry	Live births Random selection from DNBC	In-person interview; Week 17 of pregnancy (mean)	30 %	17–43 (30.0)	26.6	28.2
Iowa; 287/302; Case–control study	1987–1991	Iowa, USA	Live births, stillbirths, and elective terminations Iowa State Registry of Congenital and Inherited Disorders	Live births Random selection from Iowa birth Certificates	Telephone interview; 2.5 months after delivery (mean)	Cases 74 % Controls 55 %	15–41 (27.1)	22.2	7.6
MoBa; 184/551; Case–control analysis nested within cohort	2000–2009	Norway	Live births Norway Medical Birth Registry	Live births Random selection from MoBa	Mailed questionnaire; Weeks 15 and 30 of pregnancy	45 %	18–44 (30.0)	23.8	2.6
NBDPS; 3491/8357; Case–control study	1997–2008	10 states, USA	Live births, stillbirths, and elective terminations State birth defect registries	Live births Random selection from birth certificates; frequency matched to cases by state and date of birth	Telephone interview; 6–24 months after delivery	Varied by state: Cases 58–77 % Controls 63–73 %	13–49 (26.9)	16.3	17.3
NCL; 570/763; Case–control study	1996–2001	Norway	Live births Referral from 2 surgical centers (Oslo, Bergen) handling all cleft repair in Norway	Live births Random selection from Norway MBR	Mailed questionnaire; 14 weeks after delivery for cases and 15 weeks after delivery for controls (mean)	Cases 88 % Controls 76 %	16–44 (29.2)	31.9	11.4
Utah; 561/660; Case–control study	1995–2004	Utah, USA	Live births, stillbirths, and elective terminations Utah Birth Defects Network	Live births Random selection from Utah birth certificates; frequency matched to cases by month and year of delivery and sex of child	Telephone interview (in-person interview if telephone not available); 3–4 years after delivery	Cases 87 % Controls 85 %	15–44 (26.8)	8.0	6.5

*DNBC* Danish National Birth Cohort, *MoBa* Norwegian Mother and Child Cohort Study, *NBDPS* National Birth Defects Prevention Study (United States), *NCL* Norway Facial Clefts Study

### Alcohol exposure

#### Timing of alcohol consumption

We examined alcohol consumption during the first 3 months of pregnancy to capture the relevant exposure period for early facial development. The embryonic development of the lip and palate occurs early in pregnancy: closure of lip occurs 5–6 weeks post-conception and closure of the palate 7–10 weeks post-conception [15]. In the prospective cohort studies (DNBC, MoBa), questionnaires were administered to mothers near the end of the first trimester [week 15 for MoBa and between weeks 12–27 (mean 17) for DNBC] to ask about exposures (including alcohol consumption) up to that point in the pregnancy. In addition, the MoBa Study administered a questionnaire in the 30th week of pregnancy that repeated questions on alcohol consumption during weeks 0–12 [16]; for this analysis, we used the maximum intake reported across the two questionnaires due to evidence that maternal prenatal alcohol consumption tends to be underreported [17]. For the rest of the studies, information on alcohol intake during pregnancy was obtained retrospectively in the months after mothers gave birth. Most of these studies asked specifically about alcohol consumption during the first 3 months of pregnancy. The Iowa study asked about alcohol intake any time during the pregnancy, but had an additional question on the timing of drinking cessation that allowed us to identify women who likely drank in the first trimester.

#### Alcohol measures

In our main analyses, we used two variables to characterize binge-level drinking: the average number of drinks per sitting (no alcohol consumption, average 1–4 drinks/sitting, average 5+ drinks/sitting) and the maximum number of drinks per sitting (no alcohol consumption, never >4 drinks/sitting, ever 5+ drinks/sitting). In the first measure, women drinking an average of 5 or more drinks per sitting consumed alcohol at binge-levels, on average, each time they drank (“chronic” bingers). In the second measure, the high exposure category encompasses all of the women who ever drank 5 or more drinks per sitting, including chronic binge drinkers as well as women who reported at least one binge-drinking episode during the first trimester but whose average drinks per sitting did not exceed 4 (“periodic” bingers). We also conducted analyses to examine the average dose of alcohol and frequency of alcohol consumption together (no alcohol consumption, average 1–4 drinks per sitting during 1–2 times, average 1–4 drinks per sitting during 3 or more times, average 5+ drinks per sitting during 1–2 times, average 5+ drinks per sitting during 3 or more times). Women who reported abstaining from drinking alcohol during the first trimester (non-drinkers) served as the reference group for each of the alcohol measures. Data were standardized across studies to obtain uniform exposure and covariate variables; for example, some studies used finer categories for some variables, which were collapsed to create uniform measures. Four of the studies collected appropriate data for both of the alcohol exposures and two had data for one exposure (NCL had data on average drinks/time; DNB had maximum drinks per time), and therefore 5 of the 6 studies contributed to each of the pooled analyses.

### Statistical analysis

We used a three-step approach to the main analysis. We first estimated study-specific odds ratios (OR) and 95 % confidence intervals (CI) of the associations between the alcohol exposures and clefts in each study using multivariable logistic regression models. We then combined the data from individual studies to calculate pooled odds ratios using multivariable logistic regression adjusting for study site using a dummy variable (equivalent to fixed effect meta-analysis model) [18]. Finally, we also pooled study-specific odds ratios using random-effects meta-analysis models. The *I* ^2^ statistic was used to estimate the percentage of total variation among studies due to heterogeneity rather than chance, with a value of 0 % indicating none and higher values indicating increasing heterogeneity [19]. Separate analyses were conducted for cleft lip only (CLO), cleft lip with cleft palate (CLP), cleft palate only (CPO), as well as all cleft types combined. Infants with no additional malformations or known syndromes were classified as having “isolated clefts.” We studied infants with isolated and nonisolated clefts together to increase statistical power. In sensitivity analyses, we repeated all analyses to calculate estimates for infants with isolated clefts only. All results were adjusted for mother’s age at the child’s birth (continuous) and smoking during first trimester of pregnancy (yes/no). Further adjustment for mother’s educational level (<high school, high school, >high school) did not substantially change estimates. In analyses of alcohol dose (average drinks per time) and frequency (number of drinking episodes) together, we calculated study-specific estimates when possible (data were sparse in some studies) and pooled odds ratios using multivariable logistic regression. Analyses were conducted using Stata software [20, 21].

## Results

Control mothers in the European studies were slightly older (mean 29–30 years) than those in the American studies (mean 27 years) (Table 1). Among control mothers, smoking during the first trimester of pregnancy was less common in Utah (8 %) than the other studies (16–32 %) and low education level (<high school) was less common in the MoBa Study (2.6 vs. 6.5–28 % in the other studies). Across studies, 0.9–3.2 % of control mothers reported drinking an average of 5+ drinks per sitting and 1.4–23.5 % of control mothers reported ever consuming a maximum of 5+ drinks per sitting (Table 2). In the pooled data, 1.8 % of control mothers and 2.4 % of case mothers reported drinking an average of 5 or more drinks/sitting. When including the periodic binge drinkers in the exposure definition (maximum of 5 or more drinks/sitting), 6.7 % of control mothers and 6.3 % of case mothers were exposed.

**Table 2:** Numbers and percentages of participants by study, maternal alcohol consumption in first trimester, and infant cleft status

Study	Alcohol measure	Controls		Cleft lip only		Cleft lip with cleft palate		Cleft palate only		All clefts	
		*n*	%	*n*	%	*n*	%	*n*	%	*n*	%
*Average number of drinks per sitting*											
Iowa	None	189	64.3	23	44.2	68	64.2	83	72.2	174	63.7
	1–4	100	34.0	28	53.9	34	32.1	30	26.1	92	33.7
	5+	5	1.7	1	1.9	4	3.8	2	1.7	7	2.6
MoBa	None	349	68.8	17	63.0	53	61.6	48	84.2	118	69.4
	1–4	146	28.8	9	33.3	31	36.1	8	14.0	48	28.7
	5+	12	2.4	1	3.7	2	2.3	1	1.8	4	2.4
NBDPS	None	6356	78.4	594	76.7	1139	78.7	917	78.5	2650	78.2
	1–4	1621	20.0	161	20.8	282	19.5	233	20.0	676	20.0
	5+	135	1.7	19	2.5	26	1.8	18	1.5	63	1.9
NCL	None	527	69.5	83	60.1	147	63.1	120	61.2	350	61.7
	1–4	207	27.3	42	30.4	74	31.8	64	32.7	180	31.8
	5+	24	3.2	13	9.4	12	5.2	12	6.1	37	6.5
Utah	None	617	93.6	130	91.6	219	95.2	167	89.8	516	92.5
	1–4	36	5.5	11	7.8	10	4.4	14	7.5	35	6.3
	5+	6	0.9	1	0.7	1	0.4	5	2.7	7	1.3
Pooled	None	8038	77.8	847	74.8	1626	77.4	1335	77.5	3808	76.8
	1–4	2110	20.4	251	22.2	431	20.5	349	20.3	1031	20.8
	5+	182	1.8	35	3.1	45	2.1	38	2.2	118	2.4
	Missing	303		28		61		50		139	
*Maximum number drinks per sitting*											
DNBC	None	384	46.4	27	46.6	27	40.9	23	42.6	77	43.3
	Never >4	249	30.1	15	25.9	20	30.3	12	22.2	47	26.4
	5+	194	23.5	16	27.6	19	28.8	19	35.2	54	30.3
Iowa	None	189	64.3	23	44.2	68	64.2	83	72.2	174	63.7
	Never >4	97	33.0	27	51.9	33	31.1	27	23.5	87	31.8
	5+	8	2.7	2	3.9	5	4.7	5	4.4	12	4.4
MoBa	None	349	68.2	17	60.7	53	60.9	48	82.8	118	68.2
	Never >4	84	16.4	5	17.9	23	26.4	6	10.3	34	19.7
	5+	79	15.4	6	21.4	11	12.6	4	6.9	21	12.1
NBDPS	None	6356	77.6	594	75.9	1139	77.4	917	77.5	2650	77.1
	Never >4	1426	17.4	147	18.8	237	16.1	208	17.6	592	17.2
	5+	407	5.0	42	5.4	95	6.5	58	4.9	195	5.7
Utah	None	617	93.6	130	91.6	219	95.2	167	89.8	516	92.5
	Never >4	33	5.0	10	7.0	9	3.9	13	7.0	32	5.7
	5+	9	1.4	2	1.4	2	0.9	6	3.2	10	1.8
Pooled	None	7895	75.3	791	74.4	1506	76.8	1238	77.6	3535	76.5
	Never >4	1889	18.0	204	19.2	322	16.4	266	16.7	792	17.2
	5+	697	6.7	68	6.4	132	6.7	92	5.8	292	6.3
	missing	217		14		35		34		83	

*MoBa* Norwegian Mother and Child Cohort Study, *NBDPS* National Birth Defects Prevention Study (United States), *NCL* Norway Facial Clefts Study, *DNBC* Danish National Birth Cohort

**Fig. 1:**
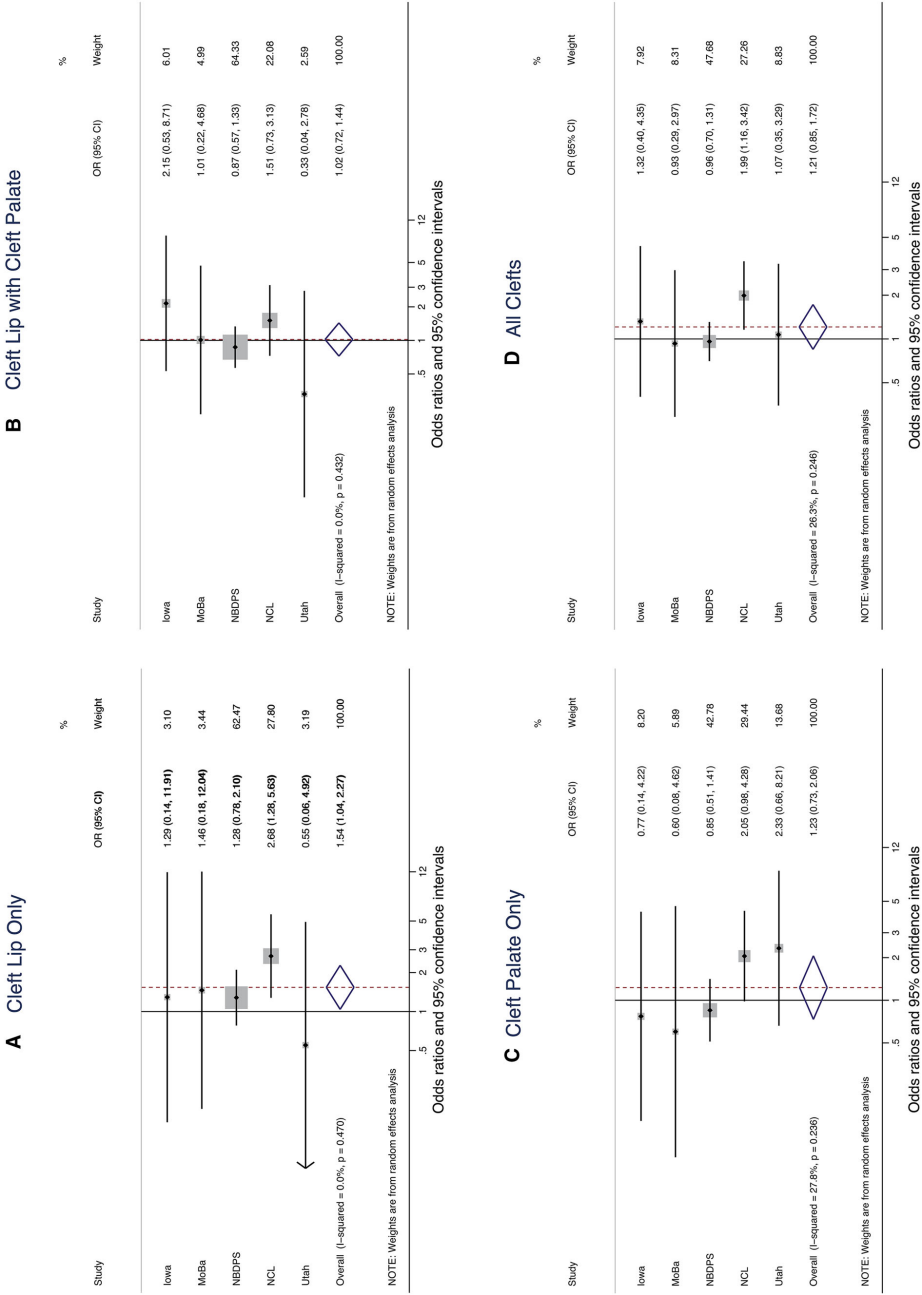
Average maternal alcohol consumption of 5+ drinks per sitting compared with no alcohol consumption in first trimester: study-specific and summary odds ratios and 95 % confidence intervals for **a** cleft lip only, **b** cleft lip with cleft palate, **c** cleft palate only, and **d** all clefts. Summary estimates were calculated using a random-effects meta-analysis model. % Weight describes the weighting each study contributed to the summary estimate. The *dots* represent study-specific odds ratios and the size of the surrounding square illustrates the weight of the study in the pooled analysis. The *horizontal lines* represent 95 % confidence intervals; if ending in an *arrow*, this indicates that the interval transcends the plot region. The *diamond* represents the summary odds ratio and 95 % confidence intervals. *MoBa* Norwegian Mother and Child Cohort Study, *NBDPS* National Birth Defects Prevention Study (United States), *NCL* Norway Facial Clefts Study

In 4 out of 5 studies, there was little evidence that mothers who drank an average of 5+ drinks per sitting had an increased risk of delivering a child with an orofacial cleft compared with non-drinkers (Table  3). The exception was the Norway Facial Clefts Study with study-specific odds ratios of 2.68 (1.28, 5.65) for cleft lip only and 2.05 (0.98, 4.27) for cleft palate only. Study-specific estimates in Iowa and Utah suggested increased risks of cleft lip with cleft palate (adjusted OR 2.15; 0.53, 8.69) and cleft palate only (adjusted OR 2.33; 0.66, 8.20), respectively, but these estimates were imprecise, with low power. In the multivariable logistic regression of pooled data, maternal consumption of an average of 5+ drinks per sitting was associated with an increased risk of infant cleft lip only (adjusted pooled OR 1.48; 1.01, 2.18). Pooled estimates from the random-effects meta-analysis were similar to, but tended to be slightly larger than, those from the pooled multivariable logistic regression (for example, random-effects pooled OR for cleft lip only = 1.54) (Fig. 1). The *I* ^2^ values indicated no evidence of heterogeneity between studies in the analyses of cleft lip only and cleft lip with cleft palate (*I* ^2^ = 0.0 %) and low levels of heterogeneity in the studies of cleft palate only (*I* ^2^ = 27.8 %) and all clefts combined (*I* ^2^ = 26.3 %).

**Table 3:** Adjusted study-specific and pooled odds ratios and 95 % confidence intervals for the association between maternal first trimester alcohol consumption (average drinks/time) and infant clefts

Study	Average number drinks per sitting	Cleft lip only		Cleft lip with cleft palate		Cleft palate only		All clefts	
		OR	95 % CI	OR	95 % CI	OR	95 % CI	OR	95 % CI
Iowa	None	1.00		1.00		1.00		1.00	
	1–4	2.15	1.17, 3.97	0.95	0.59, 1.56	0.66	0.41, 1.08	0.97	0.68, 1.39
	5+	1.29	0.14, 11.94	2.15	0.53, 8.69	0.77	0.14, 4.20	1.32	0.40, 4.35
MoBa	None	1.00		1.00		1.00		1.00	
	1–4	1.11	0.47, 2.65	1.50	0.91, 2.47	0.42	0.19, 0.90	1.00	0.67, 1.48
	5+	1.46	0.18, 12.24	1.01	0.22, 4.73	0.60	0.08, 4.75	0.93	0.29, 2.96
NBDPS	None	1.00		1.00		1.00		1.00	
	1–4	1.00	0.83, 1.20	0.93	0.81, 1.08	0.91	0.78, 1.07	0.94	0.85, 1.04
	5+	1.28	0.78, 2.10	0.87	0.57, 1.34	0.85	0.51, 1.41	0.96	0.70, 1.30
NCL	None	1.00		1.00		1.00		1.00	
	1–4	1.23	0.81, 1.86	1.23	0.88, 1.71	1.37	0.96, 1.95	1.28	1.00, 1.64
	5+	2.68	1.28, 5.65	1.51	0.73, 3.14	2.05	0.98, 4.27	1.99	1.16, 3.43
Utah	None	1.00		1.00		1.00		1.00	
	1–4	1.09	0.51, 2.31	0.57	0.26, 1.23	1.16	0.58, 2.31	0.90	0.54, 1.50
	5+	0.55	0.06, 4.80	0.33	0.04, 2.84	2.33	0.66, 8.20	1.07	0.35, 3.30
Pooled	None	1.00		1.00		1.00		1.00	
	1–4	1.08	0.93, 1.26	0.98	0.87, 1.11	0.93	0.81, 1.06	0.98	0.90, 1.07
	5+	1.48	1.01, 2.18	1.00	0.71, 1.39	1.12	0.78, 1.61	1.13	0.89, 1.44

Results were adjusted for maternal age (continuous) and smoking in first trimester (yes/no); pooled results were further adjusted for study site

*OR* odds ratio, *CI* confidence interval, *MoBa* Norwegian Mother and Child Cohort Study, *NBDPS* National Birth Defects Prevention Study (United States), *NCL* Norway Facial Clefts Study

**Fig. 2:**
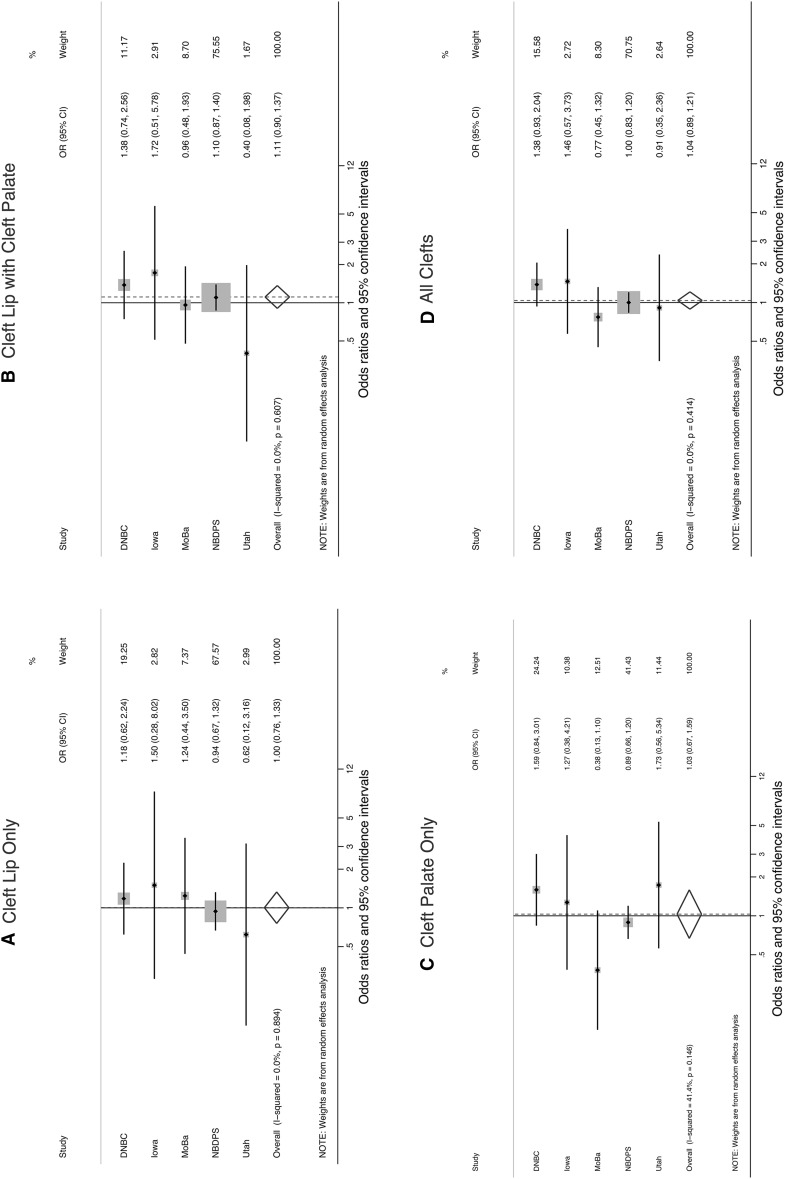
Maximum maternal alcohol consumption of 5 or more drinks per sitting compared with no alcohol consumption in first trimester: study-specific and summary odds ratios and 95 % confidence intervals for **a** cleft lip only, **b** cleft lip with cleft palate, **c** cleft palate only, and **d** all clefts. Summary estimates were calculated using a random-effects meta-analysis model. % Weight indicates the weight that each study contributed to the summary estimate. The *dots* represent study-specific odds ratios and the size of the surrounding *square* illustrates the weight of the study in the pooled analysis. The *horizontal lines* represent 95 % confidence intervals; if ending in an *arrow*, this indicates that the interval transcends the plot region. The *diamond* represents the summary odds ratio and 95 % confidence intervals. *DNBC* Danish National Birth Cohort, *MoBa* Norwegian Mother and Child Cohort Study, *NBDPS* National Birth Defects Prevention Study (United States)

There was little evidence that women who ever drank a maximum of 5 or more drinks per sitting (ever binge drinkers) had a greater risk of delivering an infant with an orofacial cleft compared with non-drinking mothers (Table 4). There were no persuasive adjusted study-specific associations, and pooled estimates were <1.10 for each cleft type in the multivariable logistic regression models of combined data. Summary estimates from the random-effects meta-analysis were similar to those from the pooled multivariable logistic regression (Fig. 2). The *I* ^2^ values indicated moderate levels of heterogeneity in the studies of cleft palate only (*I* ^2^ = 41.4 %) but none in the studies of the other 3 cleft types (*I* ^2^ = 0.0 %).

**Table 4:** Adjusted study-specific and pooled odds ratios and 95 % confidence intervals for the association between maternal first trimester alcohol consumption (maximum drinks/time) and infant clefts

Study	Maximum number drinks per sitting	Cleft lip only		Cleft lip with cleft palate		Cleft palate only		All clefts	
		OR	95 % CI	OR	95 % CI	OR	95 % CI	OR	95 % CI
DNBC	None	1.00		1.00		1.00		1.00	
	Never >4	0.90	0.46, 1.76	1.34	0.72, 2.49	0.80	0.38, 1.66	1.02	0.68, 1.53
	5+	1.18	0.62, 2.24	1.38	0.74, 2.55	1.59	0.84, 3.01	1.38	0.93, 2.04
Iowa	None	1.00		1.00		1.00		1.00	
	Never >4	2.16	1.17, 4.00	0.96	0.58, 1.56	0.62	0.38, 1.03	0.95	0.67, 1.37
	5+	1.50	0.28, 8.01	1.72	0.51, 5.76	1.27	0.38, 4.18	1.46	0.57, 3.73
MoBa	None	1.00		1.00		1.00		1.00	
	Never >4	1.27	0.45, 3.56	1.99	1.14, 3.50	0.55	0.23, 1.33	1.29	0.81, 2.04
	5+	1.24	0.44, 3.51	0.96	0.48, 1.95	0.38	0.13, 1.09	0.77	0.45, 1.32
NBDPS	None	1.00		1.00		1.00		1.00	
	Never >4	1.05	0.88, 1.27	0.90	0.78, 1.05	0.93	0.79, 1.10	0.95	0.85, 1.06
	5+	0.94	0.67, 1.33	1.10	0.87, 1.40	0.89	0.66, 1.19	1.00	0.83, 1.20
Utah	None	1.00		1.00		1.00		1.00	
	Never >4	1.13	0.52, 2.44	0.57	0.26, 1.27	1.21	0.60, 2.45	0.92	0.54, 1.56
	5+	0.62	0.12, 3.12	0.40	0.08, 1.96	1.73	0.56, 5.34	0.91	0.35, 2.36
Pooled	None	1.00		1.00		1.00		1.00	
	Never >4	1.10	0.93, 1.30	0.94	0.82, 1.08	0.88	0.77, 1.03	0.96	0.87, 1.05
	5+	1.02	0.77, 1.33	1.09	0.88, 1.34	0.96	0.76, 1.22	1.03	0.89, 1.20

Results were adjusted for maternal age (continuous) and smoking in first trimester (yes/no); pooled results were further adjusted for study site

*OR* odds ratio, *CI* confidence interval, *DNBC* Danish National Birth Cohort, *MoBa* Norwegian Mother and Child Cohort Study, *NBDPS* National Birth Defects Prevention Study (United States)

**Fig. 3:**
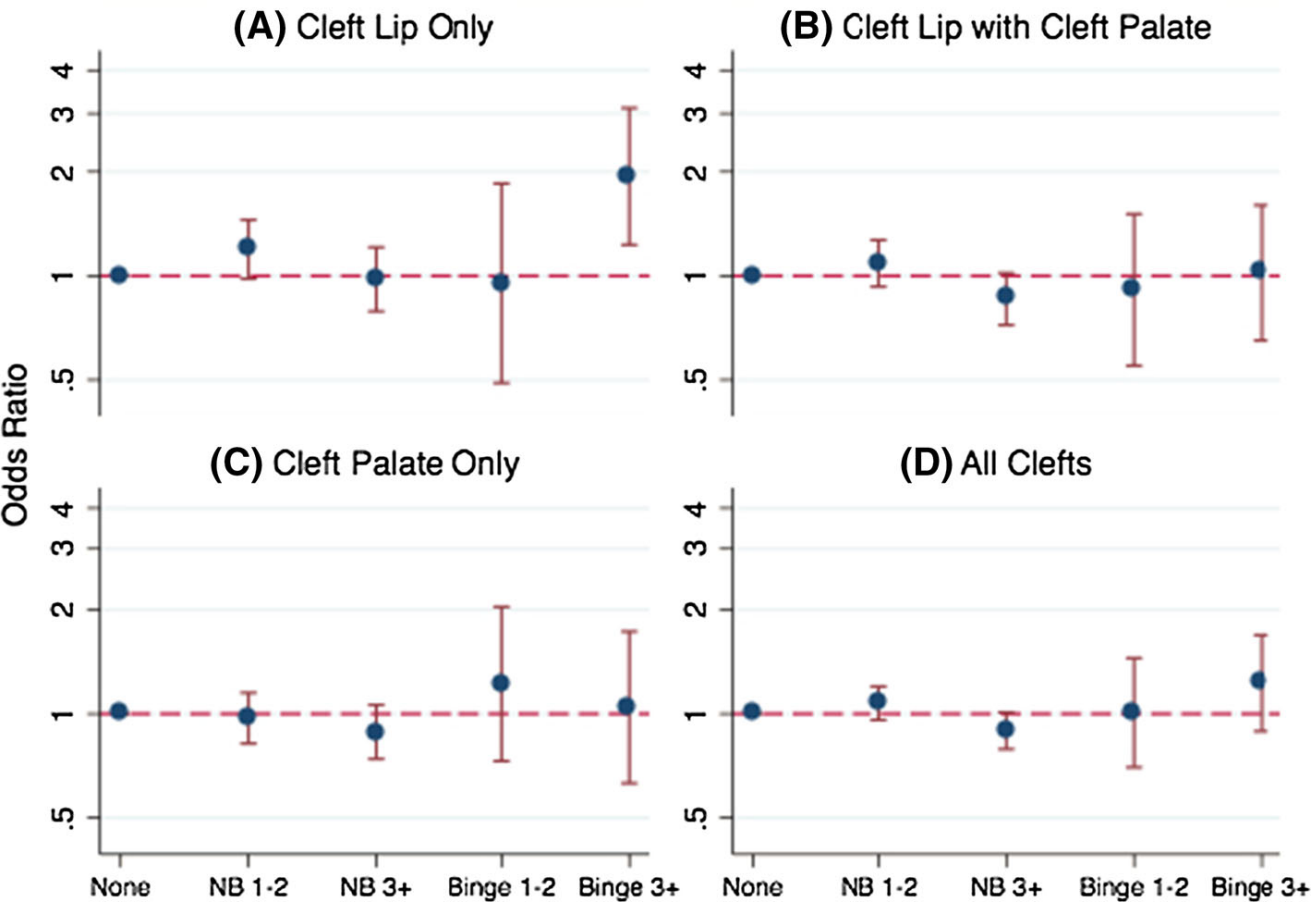
Average maternal alcohol dose and frequency of alcohol consumption in first trimester: summary odds ratios and 95 % confidence intervals for **a** cleft lip only, **b** cleft lip with cleft palate, **c** cleft palate only, and **d** all clefts. Results were adjusted for maternal age (continuous), smoking in first trimester (yes/no), and study site. The *vertical lines* represent 95 % confidence intervals. NB = non-binge drinking defined as an average of <=4 drinks/sitting; Binge defined as an average of 5+ drinks/sitting

In our analyses examining dose and frequency, 0.8 % of the total pooled control mothers drank an average of 5 or more drinks per sitting during 1–2 episodes in the first trimester and 1.0 % drank at that level 3 or more times (Table 5). Women who drank an average of 5 or more drinks per sitting who were in the highest frequency category (3 or more drinking times) had an increased risk of delivering an infant with cleft lip only compared with non-drinkers (adjusted pooled OR 1.95; 1.23, 3.11) (Table 6) (Fig. 3). For this high exposure group, the study-specific results were generally consistent, with 4 of the 5 studies exhibiting increased risk estimates (ORs ranging from 1.69 to 5.21). Drinking at this level 1–2 times was not associated with increased risk of cleft lip only (adjusted pooled OR 0.94; 0.49, 1.85).

**Table 5:** Numbers and percentages of participants by study, maternal alcohol consumption in first trimester (average drinks/sitting and number of drinking times), and infant cleft status

Study	Average number drinks per sitting and number of drinking times	Controls		Cleft lip only		Cleft lip + cleft palate		Cleft palate only		All clefts	
		*n*	%	*n*	%	*n*	%	*n*	%	*n*	%
Iowa	None	189	64.3	23	44.2	68	64.2	83	72.2	174	63.7
	Non-binge, 1–2	81	27.6	19	36.5	28	26.4	21	18.3	68	24.9
	Non-binge, 3+	19	6.5	9	17.3	6	5.7	9	7.8	24	8.8
	Binge, 1–2	3	1.0	0	0.0	1	0.9	0	0.0	1	0.4
	Binge, 3+	2	0.7	1	1.9	3	2.8	2	1.7	6	2.2
MoBa	None	349	69.9	17	63.0	53	62.4	48	84.2	118	69.8
	Non-binge, 1–2	94	18.8	9	33.3	23	27.1	7	12.3	39	23.1
	Non-binge, 3+	44	8.8	0	0.0	7	8.2	1	1.8	8	4.7
	Binge, 1–2	5	1.0	0	0.0	1	1.2	1	1.8	2	1.2
	Binge, 3+	7	1.4	1	3.7	1	1.2	0	0.0	2	1.2
NBDPS	None	6356	78.5	594	76.7	1139	78.7	917	78.6	2650	78.3
	Non-binge, 1–2	745	9.2	83	10.7	142	9.8	114	9.8	339	10.0
	Non-binge, 3+	867	10.7	78	10.1	138	9.6	118	10.1	334	9.9
	Binge, 1–2	56	0.7	4	0.5	8	0.6	7	0.6	19	0.6
	Binge, 3+	78	1.0	15	1.9	17	1.2	11	0.9	43	1.3
NCL	None	527	69.5	83	60.1	147	63.1	120	61.2	350	61.7
	Non-binge, 1–2	143	18.9	29	21.0	55	23.6	45	23.0	129	22.8
	Non-binge, 3+	64	8.4	13	9.4	19	8.2	19	9.7	51	9.0
	Binge, 1–2	17	2.2	6	4.4	9	3.9	9	4.6	24	4.2
	Binge, 3+	7	0.9	7	5.1	3	1.3	3	1.5	13	2.3
Utah	None	617	93.6	130	91.6	219	95.2	167	89.8	516	92.5
	Non-binge, 1–2	14	2.1	3	2.1	4	1.7	4	2.2	11	2.0
	Non-binge, 3+	22	3.3	8	5.6	6	2.6	10	5.4	24	4.3
	Binge, 1–2	2	0.3	1	0.7	0	0.0	2	1.1	3	0.5
	Binge, 3+	4	0.6	0	0.0	1	0.4	3	1.6	4	0.7
Pooled	None	8038	78.0	847	74.8	1626	77.5	1335	77.6	3808	76.9
	Non-binge, 1–2	1077	10.4	143	12.6	252	12.0	191	11.1	586	11.8
	Non-binge, 3+	1016	9.9	108	9.5	176	8.4	157	9.1	441	8.9
	Binge, 1–2	83	0.8	11	1.0	19	0.9	19	1.1	49	1.0
	Binge, 3+	98	1.0	24	2.1	25	1.2	19	1.1	68	1.4
	Missing	321		28		65		51		144	

*MoBa* Norwegian Mother and Child Cohort Study, *NBDPS* National Birth Defects Prevention Study (United States), *NCL* Norway Facial Clefts Study

Non-binge = average of 1–4 drinks per time; Binge = average of 5 or more drinks per time

**Table 6:** Adjusted study-specific and pooled odds ratios and 95 % confidence intervals for the association between maternal first trimester alcohol consumption (average drinks/time and number of drinking times) and infant clefts

Study	Average number drinks per sitting and number of drinking times	Cleft lip only		Cleft lip + cleft palate		Cleft palate only		All clefts	
		OR	95 % CI	OR	95 % CI	OR	95 % CI	OR	95 % CI
Iowa	None	1.00		1.00		1.00		1.00	
	Non-binge, 1–2	1.83	0.94, 3.58	0.97	0.58, 1.62	0.58	0.34, 1.00	0.89	0.61, 1.31
	Non-binge, 3+	3.50	1.39, 8.78	0.90	0.34, 2.39	1.03	0.44, 2.39	1.33	0.70, 2.52
	Binge, 1–2	–	–	0.82	0.08, 8.28	–	–	0.31	0.03, 3.03
	Binge, 3+	3.33	0.28, 39.28	4.34	0.68, 27.7	1.92	0.25, 14.45	2.90	0.57, 14.79
MoBa	None	1.00		1.00		1.00		1.00	
	Non-binge, 1–2	1.80	0.75, 4.32	1.74	1.00, 3.03	0.57	0.25, 1.30	1.26	0.81, 1.96
	Non-binge, 3+	–	–	1.15	0.48, 2.74	0.17	0.02, 1.28	0.56	0.25, 1.23
	Binge, 1–2	–	–	1.28	0.14, 11.27	1.44	0.16, 12.66	1.17	0.22, 6.15
	Binge, 3+	2.24	0.25, 20.16	0.80	0.09, 6.75	–	–	0.74	0.15, 3.69
NBDPS	None	1.00		1.00		1.00		1.00	
	Non-binge, 1–2	1.14	0.89, 1.45	1.03	0.85, 1.24	1.00	0.81, 1.24	1.04	0.91, 1.20
	Non-binge, 3+	0.89	0.69, 1.14	0.84	0.69, 1.03	0.84	0.68, 1.03	0.85	0.74, 0.98
	Binge, 1–2	0.68	0.25, 1.89	0.68	0.32, 1.43	0.84	0.38, 1.85	0.73	0.43, 1.23
	Binge, 3+	1.69	0.95, 2.99	0.94	0.55, 1.61	0.87	0.46, 1.65	1.09	0.75, 1.60
NCL	None	1.00		1.00		1.00		1.00	
	Non-binge, 1–2	1.21	0.76, 1.95	1.32	0.92, 1.91	1.39	0.94, 2.06	1.32	1.0, 1.75
	Non-binge, 3+	1.27	0.66, 2.43	1.02	0.59, 1.77	1.33	0.76, 2.33	1.19	0.80, 1.77
	Binge, 1–2	1.61	0.57, 4.54	1.62	0.70, 3.75	2.16	0.93, 5.03	1.82	0.95, 3.48
	Binge, 3+	5.21	1.76, 15.45	1.25	0.32, 4.96	1.76	0.44, 6.97	2.41	0.94, 6.13
Utah	None	1.00		1.00		1.00		1.00	
	Non-binge, 1–2	0.84	0.23, 3.10	0.61	0.19, 1.96	0.90	0.28, 2.85	0.76	0.33, 1.73
	Non-binge, 3+	1.21	0.50, 2.93	0.54	0.20, 1.42	1.31	0.58, 2.97	0.98	0.52, 1.82
	Binge, 1–2	2.01	0.18, 23.09	–	–	3.32	0.44, 24.80	1.56	0.25, 9.60
	Binge, 3+	–	–	0.48	0.05, 4.54	1.90	0.39, 9.15	0.84	0.20, 3.52
Pooled	None	1.00		1.00		1.00		1.00	
	Non-binge, 1–2	1.19	0.98, 1.45	1.09	0.93, 1.27	0.97	0.82, 1.15	1.07	0.96, 1.20
	Non-binge, 3+	0.98	0.79, 1.21	0.86	0.72, 1.02	0.88	0.74, 1.06	0.89	0.79, 1.01
	Binge, 1–2	0.94	0.49, 1.85	0.91	0.55, 1.51	1.22	0.73, 2.04	1.00	0.70, 1.45
	Binge, 3+	1.95	1.23, 3.11	1.02	0.65, 1.60	1.04	0.63, 1.73	1.23	0.89, 1.69

Results were adjusted for maternal age (continuous) and smoking in first trimester (yes/no); pooled results were further adjusted for study site

“–“indicates estimates could not be calculated because there were no exposed case mothers

*OR* odds ratio, *CI* confidence interval, *MoBa* Norwegian Mother and Child Cohort Study, *NBDPS* National Birth Defects Prevention Study (United States), *NCL* Norway Facial Clefts Study

Non-binge = average of 1–4 drinks per time; Binge = average of 5 or more drinks per time

For all analyses, results for isolated cleft malformations were similar to those reported for the combined group of isolated and nonisolated cleft malformations (data not shown).

## Discussion

In this analysis of pooled data, women who binged on average every time they drank during the first trimester had an increased risk of delivering an infant with cleft lip only compared with non-drinkers. There was however no convincing evidence of such risk for cleft lip with cleft palate or cleft palate only. Among the five studies contributing to the cleft lip only finding, the Norway Facial Cleft Study had a relatively larger study-specific risk estimate (OR 2.68) than the other studies (ORs ranging from 0.55 to 1.46) and was the only study with persuasive study-specific confidence limits. When considering both the alcohol dose and the frequency of consumption, the increased risk of cleft lip only was observed primarily among women who drank at this level 3 or more times during the first trimester. For this high exposure group, the study-specific results were more consistent, with 4 of the 5 studies exhibiting increased risk estimates (ORs ranging from 1.69 to 5.21). When examining women who ever drank at binge levels during the first trimester, including chronic binge drinkers and those who binged periodically, there was little evidence of an increased risk for any type of cleft. Maternal alcohol consumption below binge-levels was also not associated with infant cleft risk. If our findings reflect a causal relation, they suggest that a frequent and heavy level of maternal alcohol consumption was required to affect cleft risk in infants.

The timing of exposure during pregnancy is important in assessing the effects of fetal alcohol exposure. For orofacial clefts, the relevant exposure period is during the first trimester, but the precise critical period during which alcohol may influence facial development is unknown. The most likely vulnerable period is during weeks 5 through 10, when the structures forming the embryonic lip and palate fuse, but alcohol exposure earlier in gestation could affect cleft risk by disrupting epigenetic mechanisms controlling gene expression in embryogenesis [22] or otherwise affecting the cells destined to form the lip and palate structures. If the critical periods for embryonic development of the lip and palate are relatively brief, the chance that a heavy drinking episode takes place during the critical period may be low, especially if the drinking episodes are infrequent. This is consistent with our finding of increased cleft lip only risk primarily among the infants of women who drank at binge levels consistently and repeatedly during the first trimester. The frequent heavy drinking in this group may have increased the likelihood that the fetus was exposed to a high blood alcohol concentration during the critical period for embryonic lip development.

A systematic review and meta-analysis of studies of maternal alcohol consumption and orofacial clefts by Bell and colleagues [23] had null results, although they found their findings inconclusive due to heterogeneity in study design. In contrast with our use of original, individual-level study data, the Bell review extracted estimates from the published literature—an approach that can be prone to publication bias and sometimes problematic due to differences in statistical modeling, exposure and covariate definition and evaluation of confounding across studies [18]. Four studies [5, 24–26] contributed to their analysis of maternal binge drinking defined as drinking 5 or more drinks on one or more occasions in the first trimester (equivalent to our “ever binge” measure), with a combined odds ratio of 1.04 (0.87, 1.24) for cleft lip with or without cleft palate and 0.94 (0.74, 1.21) for cleft palate only. They did not examine “chronic” binge-level drinking of an average of 5 or more drinks per sitting or study cleft lip only as a separate subtype. Although cleft lip only and cleft lip with cleft palate have been traditionally studied together as one group, there is evidence that they are genetically distinct and therefore it is appropriate to analyze them separately when feasible [27, 28]. Our study-specific findings for the National Birth Defects and Prevention Study were consistent with a previous study using those data that examined maternal “periodic” binge drinking during the periconceptional period (1 month before pregnancy and the first 3 months of pregnancy) [26].

Our use of individual-level participant data from the various studies had several advantages compared with traditional meta-analysis [18, 29, 30]. We were able to use uniform definitions, coding, and cut-points for study variables and adjust for the same covariates across studies. The use of individual data allowed us to focus on binge-level drinking, which was not necessarily addressed in previous publications from these studies, and to examine cleft lip only as a distinct cleft subtype. We were, however, limited to the data collected in the studies. For example, we were unable to examine alternative definitions of binge drinking (such as 4+ drinks/sitting) because the categories used in some studies precluded this.

Pooling data increased the number of heavy alcohol drinkers available to study. Even so, binge drinking during pregnancy was rare in most of the studies and the overall percentage of exposed women was small. In particular, for our analyses examining the dose of alcohol and frequency of consumption, study-specific numbers were low for some categories of alcohol consumption. Studies with fewer exposed women contributed less to the pooled estimates as reflected by the study weights generated in the meta-analyses. Individually, many of the studies had low statistical power to examine binge-level maternal drinking and risk of clefts, resulting in study-specific estimates with wide confidence intervals that could not exclude the possibility of strong associations. Although we found little evidence of heterogeneity across the studies for the various alcohol measures and cleft categories, this may be due to the general lack of precision for many of the study specific estimates. The differences in the prevalence of self-reported binge drinking across studies probably reflect true variation in alcohol-use patterns in different study settings and time periods, but may also be due to reporting factors related to awareness of alcohol-related fetal harm or social stigma against drinking in pregnancy.

Many previous studies have examined isolated clefts separately, and there has been discussion in the orofacial cleft research on whether cases with associated anomalies should be included in etiologic studies [31]. We found little difference in results for isolated cleft malformations and those for the combined group of isolated and nonisolated clefts. Depending of the dose and timing of maternal alcohol consumption during pregnancy, alcohol could cause a variety of teratogenic effects in both nonsyndromic and syndromic cases. Children diagnosed with fetal alcohol syndrome sometimes have other anomalies suspected to be related to alcohol exposure, including orofacial, heart, kidney, and limb and joint malformations [32].

Recall bias is a common concern in retrospective case–controls studies. After giving birth to a healthy infant, control mothers may have been more likely than mothers of affected children to admit drinking alcohol during pregnancy. This would tend to underestimate the association between maternal prenatal alcohol consumption and infant orofacial clefts. Conversely, the association would be overestimated if mothers of cases were more likely to remember past drinking, perhaps in an effort to explain the occurrence of the malformation. For example, we cannot rule out that recall bias may have led to bias away from the null in the Norway Facial Clefts Study, which had the largest study specific estimate for cleft lip only risk among women drinking an average of 5+ drinks/sitting. For the case–control studies nested within cohorts, information on alcohol and other exposures was collected prospectively, before the birth of the child, thus avoiding potential recall bias. Although all of the studies were population-based, participation rates varied, and there may have been selection bias if heavy drinking case mothers were less likely to participate than heavy drinkers selected as controls. Women with fewer economic resources, lower education or higher stress may have been unable or reluctant to participate in the studies because of the time and effort required, particularly in the prospective cohort studies (which required longer follow up and completion of several study questionnaires). There was some evidence for this in the MoBa cohort; participants were less likely to be younger (<25 years) or smoke cigarettes, and more likely to be married, have higher education, and take multivitamin and folic acid supplements compared with all women giving birth in Norway during the enrollment years [33].

Our pooled study population included relatively few women of low socioeconomic status and therefore may not have included the women and children most susceptible to harm from prenatal alcohol exposure. Poverty may increase vulnerability to alcohol-related birth defects and other adverse birth outcomes through social and behavioral risk factors such as maternal undernutrition, psychological or physical stress, smoking or other substance abuse [34]. In addition, we did not take into account genetic susceptibility defined by maternal or fetal alcohol metabolizing genes, which could influence the peak alcohol concentration experienced by the embryo or fetus and therefore affect cleft risk. One report found that maternal binge-level drinking was associated with an increased risk of infant clefts only in mothers and children who carried the *ADH1C* haplotype associated with reduced alcohol metabolism, although these results were limited by the small numbers of heavy drinkers across haplotype groups [35].

In summary, using pooled data from five studies, we found that maternal alcohol binge drinking (average of 5+ drinks) was associated with an increased risk for one of the cleft subtypes, cleft lip only, in offspring. Women who drank at this level 3 or more times in the first trimester had a nearly twofold increased risk of having a child with cleft lip only compared with non-drinkers. Less frequent binge drinking or drinking alcohol at non-binge levels was not associated with an increased risk of any type of cleft. If causal, these findings suggest that repeated heavy prenatal maternal drinking may affect cleft lip only risk.
